# Wearable Activity Trackers That Motivate Women to Increase Physical Activity: Mixed Methods Study

**DOI:** 10.2196/48704

**Published:** 2023-12-14

**Authors:** Neil E Peterson, Danielle A Bate, Janelle LB Macintosh, Corinna Trujillo Tanner

**Affiliations:** 1 College of Nursing Brigham Young University Provo, UT United States; 2 Canyon View Medical Springville, UT United States

**Keywords:** physical activity, women, motivation, wearable activity trackers, mobile health, mHealth, self-determination, mobile phone

## Abstract

**Background:**

Physical inactivity is a significant public health concern, particularly among women in the United States. Wearable activity trackers (WATs) have been proposed as a potential solution to increase awareness of and engagement in physical activity (PA). However, to be effective, WATs must include features and designs that encourage daily use.

**Objective:**

This study aims to explore the features and designs of WATs that appeal to women and determine whether devices with these attributes are effective motivators for women to be physically active.

**Methods:**

A mixed methods study guided by the self-determination theory was conducted among 15 women. Participants trialed 3 WATs with influence in their respective accessory domains: Apple Watch for the wrist; Oura Ring for the finger; and Bellabeat Leaf Urban for multiple sites (it can be worn as a bracelet, necklace, or clip). Participants documented their daily PA levels and rated their satisfaction with each device’s comfort, features, and motivational effect. Focus groups were also conducted to gather additional feedback and experiences within the a priori areas of comfort, features, and motivation.

**Results:**

Behavioral Regulation in Exercise Questionnaire–2 scores indicated that most participants (14/15, 93%) were motivated at baseline (amotivation score: mean 0.13, SD 0.45), but on average, participants did not meet the national minimum PA guidelines according to the self-reported Physical Activity Vital Sign questionnaire (moderate to vigorous PA score: mean 144, SD 97.5 min/wk). Mean WAT wear time was 16.9 (SD 4.4) hours, 19.4 (SD 5.3) hours, and 20.4 (SD 4.7) hours for Apple Watch, Bellabeat Leaf Urban, and Oura Ring, respectively. During focus groups, participants reinforced their quantitative ratings and rankings of the WATs based on personal experiences. Participants shared a variety of both activity-related and non–activity-related features that they look for in a motivating device. When considering what the ideal WAT would be for a woman, participants suggested features of (1) comfort, (2) extended battery life, (3) durability, (4) immediate PA feedback, (5) intuitive PA sensing, and (6) programmability.

**Conclusions:**

This study is the first to specifically address women’s experiences with and preferences for different types of WATs. Those who work with women should realize how they view WATs and the role they play in motivation to be active.

## Introduction

### Background

Women are more likely to enjoy optimal health when they meet the physical activity (PA) recommendations, which include the integration of leisure-time exercise and reduction of sedentary behavior. The US Department of Health and Human Services recommends adults to achieve the equivalent of 150 to 300 minutes of moderate intensity exercise in a week [1]. Meeting these PA guidelines reduces the risk of developing chronic conditions such as diabetes, dementia, cancers, cardiovascular disease, and depression [2-4]. PA decreases the severity of existing illness and considerably reduces the risk of mortality from any cause [5-7]. Despite the health benefits of regular PA, most adults’ PA levels remain insufficient [1]. Americans experience an excessive amount of sedentary behavior, averaging 9.5 hours per day [8]. Just more than half (1718/3305, 51.98%) of American women get the recommended amount of PA [9], and worldwide, women of all ages are less physically active than their male counterparts [7,10]. Despite decades of research efforts and the development of national PA guidelines, insufficient PA is a persistent problem that accounts for US $117 billion in health care costs annually [11].

The reason that women engage in less PA than men is not well understood. Studies have compared the types of activities, rewards, or pressures that motivate women and men to be physically active [12-14] and studied environmental, demographic, and psychosocial barriers to PA [15,16]. Results reveal various limiting factors to PA among women. For example, time constraints are a barrier to PA once women transition from school to the workplace and begin fulfilling multiple roles and responsibilities [17,18]. Lack of intrinsic motivation to replace sedentary behavior with PA is also a limiting factor [15,16].

Wearable activity trackers (WATs)—devices that monitor and incentivize activity—may motivate women to monitor and make PA a part of their daily routine [19,20]. WATs provide practical PA data (step counts, calories burned, and active time), and most give personalized prompts to stand or move based on daily goals and progress. In a 2019 Gallup poll, one-third of Americans reported having worn a WAT or having tracked their health statistics on a mobile app before, and most current or former users of WATs reported finding them “very” or “somewhat” helpful [21]. Similarly, recent studies have shown that individuals who wear WATs have increased self-awareness of PA and acknowledge that trackers may be a motivational tool to improve PA behaviors [22,23]. Wearing WATs daily optimizes their effectiveness and leads to sustained PA behaviors [24-26]. Thus, ideal WATs should have features and designs that encourage daily use.

Most WAT users are women [21]. Despite the technical advances of WATs, some women describe them as bulky, unattractive, and uncomfortable, often resulting in disuse and abandonment [22,23,27]. In recent years, developers have released several new styles of WATs such as necklaces, rings, earrings, bracelets, and concealable clips. With these variations being available, WATs may be an untapped resource for motivating women to be active. Considering the value of PA and WATs’ potential to improve PA motivation, it is important to learn what types of WATs women are willing to adopt. The purpose of this study was to explore the features and designs of WATs that appeal to women and determine whether devices with these attributes are effective motivators for women to be physically active. This study is the first to specifically address women’s experiences in using and preferences for different types of WATs.

### Theoretical Frameworks

According to a psychological framework [28], men and women exit young and “emerging” adulthood and enter the “established adulthood” stage of life between the age of 30 to 45 years. During this stage, adults experience the intersection of developmental tasks, which results in more securely established roles and a great number of responsibilities. Adults in this period of life are more likely to have committed to an occupational path, entered and maintained a long-term romantic relationship, and begun having children [28]. Hypothetically, women in this phase of adulthood would also have stable PA patterns compared with those in early life stages, when changes in routine are generally more common. This study intended to capture the effects of WATs on PA motivation during and after this phase of established adulthood; hence, one of our inclusion criteria is being aged at least 30 years.

An intended outcome of wearing WATs is enhanced motivation and PA, but this result may not be true for everyone. Self-determination theory (SDT) offers a plausible explanation. According to SDT, individuals have an inherent propensity for one of the four types of self-regulation: (1) external, (2) introjected, (3) identified, and (4) intrinsic [29-31]. These exist on a continuum. How individuals experience motivation depends on their self-regulation. Those with external regulation experience motivation through rewards, pressures, or punishments separate from the activity and imposed by an outside entity. Those with introjected regulation also feel motivated by pressures or rewards separate from the activity itself, but these are self-induced, for example, the desire to avoid guilt and anxiety or to enhance self-esteem. Next on the continuum is identified regulation, wherein individuals feel motivated by the personal importance or conscious valuing of an activity. Finally, those with intrinsic regulation feel motivated by their autonomous enjoyment for activity [30]. Amotivation or the lack of any intention or value for activity is included in SDT [30]. According to SDT, WAT prompts and incentives would appeal to external regulators, whereas activity data would fuel introjected and identified regulators; each would have little bearing on intrinsic regulators. Thus, individuals wearing WATs experience a spectrum of motivational effects.

## Methods

### Ethical Considerations

Before the commencement of this study, human subjects research ethics review was completed, and approval was given by the researchers’ Brigham Young University Institutional Review Board (X2021-117). Participation was voluntary and participants could withdraw at any time. Compensation was prorated based on the number of stages completed. Participants were compensated with web-based retailer shopping gift cards worth US $10, US $20, US $40, and US $80 for trialing 1, 2, or 3 activity trackers and participating in the web focus group, respectively; thus, a total compensation of US $150 was possible for completing all 4 stages. Participants were assigned code numbers for all data entry purposes. The participant-to-code number list was then destroyed after all the study data had been collected. For participant protection, quantitative data are reported in aggregate, and qualitative focus group statements are deidentified.

### Sample and Setting

After receiving the institutional review board approval, volunteers were recruited via email and word of mouth and then screened for eligibility. Inclusion criteria were iPhone ownership (because iPhone was necessary for 1 of the trackers to function) and age of at least 30 years to meet the definition of “established adulthood.” The exclusion criterion was the presence of a condition or injury that altered body mechanics and did not allow for normal, everyday activities. For example, this would exclude women in their third trimester of pregnancy and those requiring acute use of crutches for a lower extremity injury. Baseline PA level was not a condition for participation.

Study participants used 3 settings for this study. The first was a university laboratory for in-person encounters. The second was Zoom (Zoom Video Communications), a web video chat portal where participants attended a focus group meeting. The third was Qualtrics (Qualtrics International Inc), a web survey platform where participants answered questionnaires throughout the study period.

### Instruments

#### Behavioral Regulation in Exercise Questionnaire–2

Researchers determined the participants’ baseline exercise motivation using Behavioral Regulation in Exercise Questionnaire–2 (BREQ-2), a tool based on SDT. BREQ-2 is a 19-item questionnaire that helps identify the types of regulation underlying adults’ engagement in PA: external, introjected, identified, and intrinsic. In addition, BREQ-2 includes an assessment of amotivation. Each question, ranging from 0 to 4 on a Likert scale, gives a score on each of these 5 categories of regulation. BREQ-2 is reliable, with Cronbach α ranging from .73 to .90 on all subscales [32,33]. Another study conducted a comprehensive review and found that BREQ-2 has good construct validity, in a manner consistent with SDT [34]. Therefore, BREQ-2 is an appropriate tool for determining where participants lie on the self-determination continuum regarding PA.

#### Physical Activity Vital Sign

Physical Activity Vital Sign (PAVS) is a validated, 2-item questionnaire used for assessing if an individual meets the PA guidelines. The questions are (1) “On average, how many days per week do you participate in moderate to vigorous physical activity (e.g., a brisk walk)?” and (2) “On average, how many minutes per day do you perform physical activity at this level?” When multiplied, these 2 numbers estimate the average number of minutes of PA per week [35].

#### Activity Trackers

##### Overview

Participants trialed 3 WATs that have the greatest influence in their respective accessory domains: Apple Watch (Watch) for wrist; Oura Ring (Ring) for finger; and Bellabeat Leaf Urban (Leaf) for multiple sites, which can be worn as a bracelet, necklace, or clip anywhere. The Watch and Ring were similar in price range, whereas the Leaf was approximately one-third of the cost. All of them were water resistant and tracked similar data such as daily PA minutes, steps taken, and calories burned. However, each offered the following unique designs and features.

##### Apple Watch

Although PA data are viewable in the smartphone app, the Watch’s face lets users view most data in real time on the device it || elf. The Watch updates users about their PA progress and prompts them to move using messages and vibration. In addition, PA progress is shareable among other Watch users. Numerous wrist bands are available for the Watch; participants had several options to choose from based on style and comfort preferences. The Watch’s battery typically lasts for up to 18 hours. Charging takes up to 120 minutes.

##### Oura Ring

The Ring is unique for its finger placement, minimalist design, and 4- to 7-day battery life. Charging the Ring also takes up to 120 minutes. It adjusts the exercise targets as the wearer progresses and reaches goals and prompts users to move via smartphone notifications. PA data are viewable in the smartphone app.

##### Bellabeat Leaf Urban

The Leaf was designed for women and by women and is intended to resemble a piece of jewelry rather than a WAT. It can function as a bracelet, necklace, or clip. The Leaf’s wrist bands are exchangeable but currently have fewer available options than the Watch. It is nonrechargeable, but the replaceable battery lasts for up to 6 months. Replacement batteries are inexpensive and widely available. The Leaf prompts users to move using vibration and smartphone notifications. PA data are viewable in the smartphone app.

#### Qualitative Semistructured Interview Guide

The following open-ended questions guided the focus group discussions:

1. What were your impressions about the Apple Watch?
2. What were your impressions about the Bellabeat Leaf?
3. What were your impressions about the Oura Ring?
4. If you were to design your ideal activity tracker, what features would be the most important to you?

### Procedures

Participants were organized into 5 groups; each group began on a different study start date. During their first encounter with participants, researchers obtained informed consent, administered BREQ-2, collected demographic data, and measured participants’ height and weight using a stadiometer and digital scale. Height and weight were measured without shoes, in duplicate, and averaged. In addition, participants in each group completed the PAVS questionnaire, obtained their first WAT, and watched a short informational video summarizing its functionality. Participants were asked to wear the device as often as possible during the following week and record their daily PA data in the data collection form provided. Each group member trialed the same WAT, but each group trialed the WATs in random order to control for the serial position effect. After 1 week, participants returned the devices and data collection forms; repeated PAVS; and completed a survey asking them to rate their satisfaction with the device’s comfort, features, and motivational effect. They performed this process thrice until they had trialed and rated all 3 trackers.

Participants then ranked the devices (first, second, and third) based on comfort, features, motivational effect, and overall preference. Finally, researchers led 2 recorded focus group discussions, where participants answered open-ended questions regarding the devices.

### Data Analysis

This study used a mixed methods design. Quantitative data from demographics and BREQ-2 questionnaires were evaluated using simple descriptive statistics with univariate analysis. SPSS (version 25.0; IBM Corp) was used to analyze device data (daily min of moderate to vigorous PA [MVPA], step counts, and active calories burned) and self-reported PAVS. Device data were omitted for days on which the device was worn for <10 hours or if the number of hours worn was unknown owing to participant error in completing the data collection form. A comparison of quantitative factors across the devices was performed using 1-way ANOVA, including PAVS, survey data (device ratings and rankings), and data from collection forms (calories burned, steps, and MVPA). Researchers used Zoom transcriptions of the recorded focus group meetings for descriptive, thematic, qualitative data analysis using a priori themes of *comfort*, *features*, and *motivation*.

## Results

### Descriptive Statistics

A total of 15 female participants aged between 33 and 69 (median 45) years joined and completed the WAT trials and all the surveys. A participant did not attend the focus group meeting owing to a scheduling conflict. The participants’ BMI ranged from 21.4 to 40.6 (median 24.9) kg/m². A participant declined the weight assessment. Most participants were White (14/15, 93%), were married (12/15, 80%), and had a bachelor’s degree education or higher (14/15, 93%). Two-thirds of the participants (10/15, 67%) were employed. The BREQ-2 scores indicated that most participants (14/15, 93%) were motivated at baseline (amotivation score: mean 0.13, SD 0.45). They had internal motivation (identified regulation score: mean 3.07, SD 0.75; intrinsic regulation score: mean 3.02, SD 0.84) and were less motivated by outside rewards (external regulation score: mean 0.70, SD 0.6; introjected regulation score: mean 1.78, SD 1.01). Refer to Table 1 for full demographic details and BREQ-2 results. Participants’ baseline reported PAVS scores ranged from 0 to 360 minutes of MVPA per week, with an average of 144 (SD 97.5) minutes (refer to Table 2 for all PA statistics). Correlation analysis showed a positive relationship between those with intrinsic motivation and their baseline PAVS (*r*=0.674; *P*=.006), which supports SDT. We also saw a positive correlation between baseline PAVS and PAVS during the weeks with each device (Watch: *r*=0.854; *P*<.001; Leaf: *r*=0.880; *P*<.001; Ring: *r*=0.884; *P*<.001).

**Table 1:** Demographics and Behavioral Regulation in Exercise Questionnaire–2 (BREQ-2) results (N=15).

			Values
**Demographics**			
	Age (y), mean (SD)		45.53 (9.2)
	BMI (kg/m²), mean (SD)		26.96 (5.3)
	Number of children, mean (SD)		4.53 (2.9)
	**Education level, n (%)**		
		Some college or no degree	1 (7)
		Bachelor’s degree	9 (60)
		Master’s degree	4 (27)
		Professional or doctorate degree	1 (7)
	**Relationship status, n (%)**		
		Never married	1 (7)
		Married	12 (80)
		Widowed	1 (7)
		Divorced	1 (7)
	**Employment, n (%)**		
		Homemaker	5 (33)
		Employed part time	3 (20)
		Employed full time	7 (47)
	**Annual household income (US $), n (%)**		
		25,000-50,000	1 (7)
		50,000-100,000	5 (33)
		100,000-200,000	8 (53)
		>200,000	1 (7)
**BREQ-2 score, mean (SD)**			
	External regulation		0.70 (0.60)
	Introjected regulation		1.78 (1.01)
	Identified regulation		3.07 (0.75)
	Intrinsic regulation		3.02 (0.84)
	Amotivation		0.13 (0.45)

**Table 2:** Physical activity descriptive statistics.

Daily average	Apple Watch, mean (SD)	Bellabeat Leaf Urban, mean (SD)	Oura Ring, mean (SD)
Hours worn	16.92 (4.43)	19.40 (5.27)	20.44 (4.77)
Min of MVPA^a^	31.57 (30.28)	105.53 (106.67)	48.68 (40.38)
Active calories	568.89 (231.40)	426.63 (326.25)	455.05 (230.88)
Steps	8509 (4362)	11,025 (8395)	10,795 (5000)

^a^MVPA: moderate to vigorous physical activity.

### Activity Data

Participants recorded the daily number of hours they wore a device; they also recorded the MVPA, steps and active calories recorded by each device daily. At baseline, participants’ self-reported PAVS was 144 (SD 97.36) minutes of MVPA per week. Participants’ average self-reported PAVS were 156.27 (SD 174.50) minutes for the Watch, 129.73 (SD 109.97) minutes for the Leaf, and 134 (SD 97.07) minutes for the Ring. To identify whether the devices’ activity data coincided with the reported PAVS, weekly MVPA minutes were calculated. The average weekly MVPA minutes recorded by the devices were 230.13 (SD 211.98) minutes for the Watch, 744.93 (SD 746.68) minutes for the Leaf, and 424.64 (SD 352.24) minutes for the Ring. A participant did not record her MVPA minutes for the week she wore the Ring; therefore, a sample size of 14 was used in calculating the average weekly MVPA for the weeks the Ring was worn. Although all related PAVS and weekly MVPA averages are dissimilar, the largest discrepancy exists between Leaf PAVS and MVPA data.

### Device Ratings, Rankings, and Qualitative Feedback

#### Apple Watch

**Figure 1:**
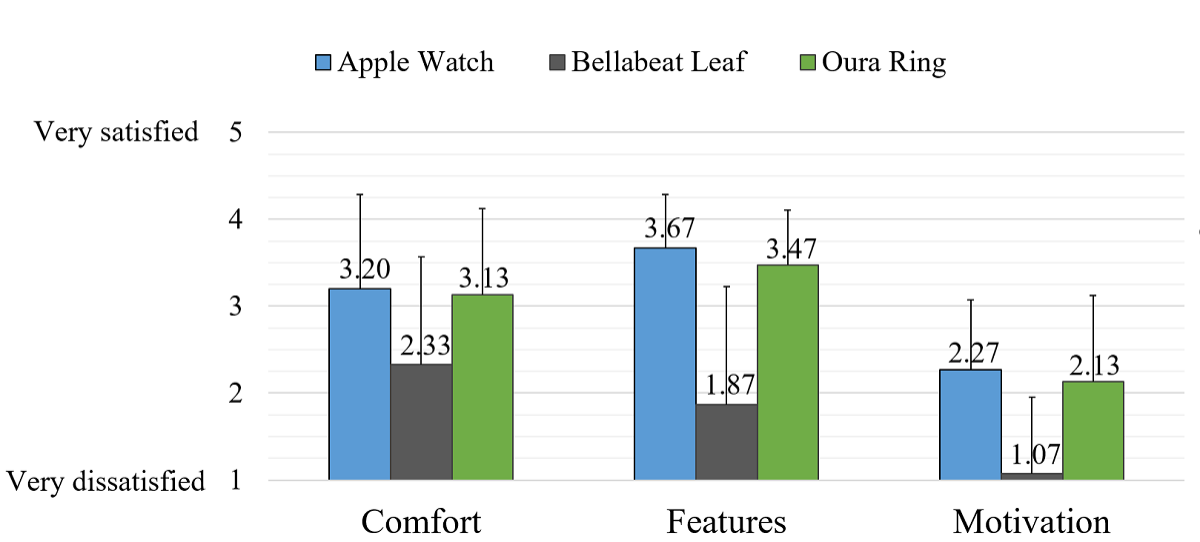
Mean satisfaction ratings. At the end of each week, the participants were asked to rate how satisfied they were with each device’s comfort and features and motivation provided by the device to be active. The rating scale ranged from 1 (very dissatisfied) to 5 (very satisfied). Above each bar is the mean score for the device, and the error bar depicts the SD.

**Figure 2:**
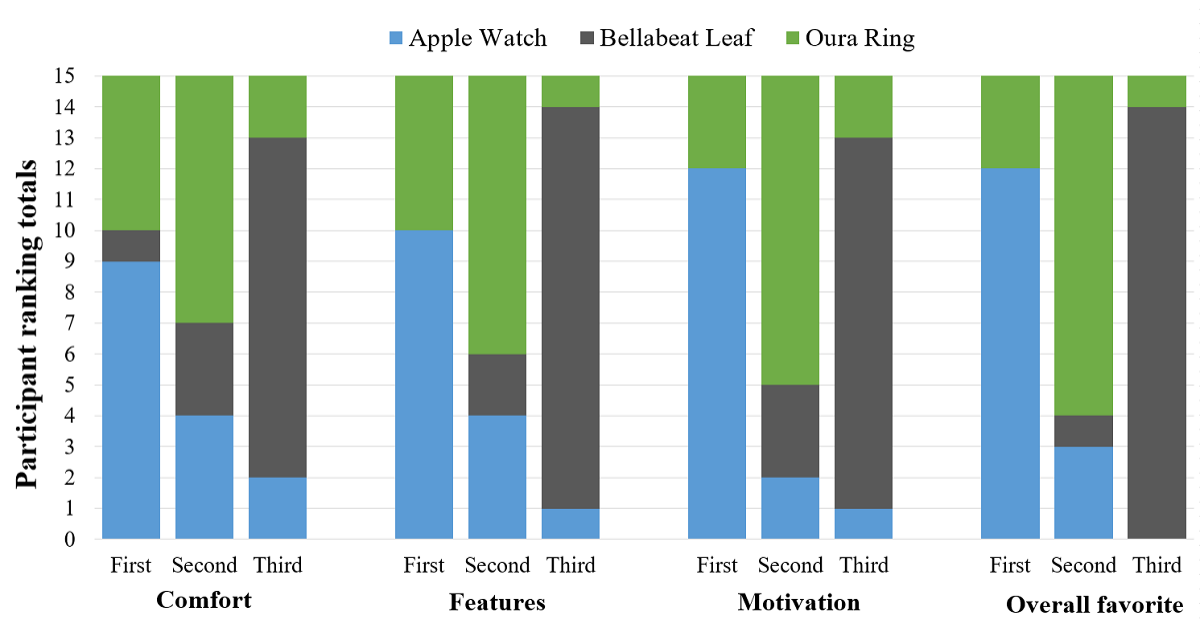
Cumulative device rankings according to category. The 15 participants were asked to rank the 3 devices (first, second, or third) on each of the following categories: comfort of the device, features the device offers, motivation provided by the device to be active, and overall favorite.

Participants were generally satisfied with the comfort and features but less so with the motivational effect of the Watch; however, they gave it high ratings in each category (Figures 1 and 2).

##### Comfort

The Watch was the most comfortable device for most participants (9/15, 60%):

I like wearing a watch...It’s more comfortable for me.

A participant commented the following:

The strap was pretty comfortable.

However, another participant said the following:

I felt like it was really heavy on my wrist and yeah I just didn’t really like it.

##### Features

The Watch’s features were ranked the highest (10/15, 67%). A participant stated the following:

I like the Apple Watch because I can see the data right there. With the Ring and bracelet [Leaf], I always had to go to my phone and look it up.

Another participant stated the following:

I liked that it combined other features besides just fitness...[it] has so many other features that I use way more than even the fitness tracker.

However, several participants (3/15, 20%) noted that the Watch did not detect all PA. A woman expressed the following:

I found it was really hard in tracking my exercise rings...I can go for a walk...and it’d be like “hey you did 4 minutes” for the 20 to 30 minutes that I was actually out doing things...So to me that was hard because it didn’t read as accurately. Whereas with the ring and the leaf, they picked it up easier.

The short battery life of the Watch was the least desirable feature. A participant said the following:

Having to charge it as often as you had to was difficult.

##### Motivation

Most participants (12/15, 80%) said that the Watch was the most motivating device. A participant said the following:

I did like watching the rings fill in...it was fun to meet my goals and be able to see it...

Another participant added the following:

It made me motivated to really work harder and do better, and keep track of it.

A third participant agreed:

I really did appreciate...the rings and I was highly motivated to try and close them every day, I was a little surprised that that’s all it took.

#### Bellabeat Leaf

Participants generally disliked the Leaf. Its average ratings for comfort, features, and motivation were the lowest among the 3 devices.

##### Comfort

Participants said that the Leaf was the least comfortable device (11/15, 73%). A woman said the following:

I could not wait until I was done with the Bellabeat because I could not stand wearing it. I was counting the days.

Another participant agreed:

It was difficult to wear and difficult to put on...When I tried to wear it around my neck, it was heavy...so I didn’t wear it as a necklace.

##### Features

The Leaf had few desirable features and was ranked last (13/15, 87%). The most common complaints were lack of PA sensing and inaccurate tracking. Participants had to manually input the type of workout and time spent in doing it. All (15/15, 100%) agreed this was cumbersome. A woman stated the following:

If I have to put it in, I might as well just get a piece of paper and write down what I did.

Regarding accuracy, a woman stated the following:

I also found it somewhat inaccurate on the number of steps. On one day I had done some hiking, it said I had done the least amount of steps which I didn’t think was right.

However, valued features of the Leaf were its versatility, esthetics, and battery life. A participant noted the following:

I appreciated that there were multiple ways you could wear it...I did try it as a clip...It was hard to know how effective it is in all the different positions...but I did kind of like that there were alternatives.

Another participant stated the following:

I thought it was the most aesthetically pleasing of the three devices...but the hassle of trying to wear it outweighed any aesthetic value.

Perhaps the most pleasing feature of the Leaf was its battery. Regarding charging, a woman said the following:

It was amazing that I didn’t have to think about that at all.

Another participant agreed:

I just left the Bellabeat on and never took it off...The fact that it had a battery, and not having to charge it, was the best part about it.

##### Motivation

The Leaf was also the least motivating among the devices (13/15, 87%). None of the participants (0/15, 0%) made any comments specifically regarding the Leaf’s motivational effects in the focus group meetings.

#### Oura Ring

The Ring had similar ratings to those of the Watch regarding comfort, features, and motivation. However, participants generally ranked it second to the Watch in all areas.

##### Comfort

Although it ranked high, initially, the Ring was uncomfortable. A participant stated the following:

I really liked the Ring. Out of all three I liked the ring best. Even though it took a while because it was kind of bulky, by the end I felt like it was comfortable and I really enjoyed it.

##### Features

Positive features were battery life, placement on a finger, and sleep tracking. Participants could wear the Ring day and night for several days before recharging. A participant said the following:

I liked that I didn’t have to charge it as often. Especially if I wanted to use it at night to track my sleep, I really liked that the battery lasted on it for a long time.

In general, the women thought that wearing the tracker on a finger was convenient; a woman stated the following:

I like that you didn’t have to take it off or do anything...I liked that. You could jump in the pool and you can wash your hands.

The Ring is known for its accurate sleep tracking capability, and this was a feature that all women (15/15, 100%) enjoyed:

I did like that I could see the REM and sleep information...I thought that was interesting.

Although participants liked some features, they complained about its size. A participant said the following:

It seemed more like a man ring. It was very masculine for me. In fact, someone asked me if I was wearing my husband’s ring. I feel like it could be a little more dainty.

A woman thought that the Ring could look and feel more similar to jewelry, saying it felt “cheap - like something that you would get out of one of the quarter station things at the grocery store.”

Participants had mixed reviews regarding the accuracy of the Ring:

It did seem like it tracked my steps more accurately than the Bellabeat...I do about the same thing every week...so my steps were more similar to the Apple watch with the ring than with the Bellabeat.

Another participant was more skeptical:

I think there was a real inaccuracy with the pedometer. It just was really off.

The Ring was the only device that categorized PA into high, medium, and low levels. A woman enjoyed this feature:

The high-medium-low activity made it nice to see how active I am. I move, but I don’t move enough to raise my heart rate.

Participants could view PA data via the mobile app in real time. However, some women missed having PA data displayed on the device itself. A woman stated the following:

The only thing I didn’t like is that you couldn’t see your activity immediately like you can on the watch.

##### Motivation

Most participants (10/15, 67%) ranked the Ring as the second most motivating device. A participant shared the following:

When it came to activity during the day, I had to check my phone and didn’t really pay attention to it.

Another participant thought that the Ring was motivating:

It made me more aware. I’m all about “it doesn’t happen unless you measure it.” If you’re not self-aware, how can you address it and make changes?

#### Ideal Device for Women

At the end of the focus group, participants were asked to describe the qualities that would make up their ideal WAT. Participants reported that the ideal WAT for women should (1) be lightweight, petite, and sufficiently comfortable to wear during sleep; (2) have a battery life that lasts for >1 day; (3) be durable and water resistant, so that it can endure all types of activities; (4) provide real-time PA feedback; (5) sense and track PA and workouts intuitively; and (6) be highly programmable, so that the user can adjust the goals and alerts. Some participants shared that the WATs were not motivating “enough to make a significant difference.” However, those who reported feeling motivated by a device agreed that the visual feedback from the Watch and the gamification of its activity rings were motivating. Thus, visual PA data on the device itself and gamification may be key features in affecting PA motivation.

## Discussion

### Principal Findings

The purpose of this study was to explore the aspects of WATs that appeal to women and whether these devices motivate them to be physically active. Overall, the Watch and Ring provided satisfactory comfort and features for the women (Figure 1). Despite its versatility and potential, the Leaf was the least preferred among all devices and did not garner any first-place ranking outside the comfort category (Figure 2). No device provided additional motivation to be active; however, this study’s participants already had high *identified* and *intrinsic* motivation according to SDT. In this small sample, self-reported PA via PAVS was not significantly different among devices.

This study supports the idea that the features and functionality of WATs have an impact on device adoption. These findings agree with those of previous study that convenience features—such as real-time feedback and intuitiveness—have a positive influence on using WATs, whereas high-maintenance functionality and frequent charging of devices are barriers to daily use [27,36,37]. Participants in this study valued the Watch’s functionality, but the need to charge it frequently and remember to wear it daily were impediments to its daily use. The long battery lives of the Ring and Leaf allowed participants to wear them for several days continuously and was the advantage of these devices. Replacing the Leaf’s battery requires disassembling the device, and although this was not necessary during this study, doing so may have a potential negative impact on the Leaf’s use.

Findings from this study align with those of previous WAT studies in that comfort is a feature that greatly influences adoption of WATs [27,36,37]. Comfort is clearly a priority for women and was among the first qualities mentioned when asked about what comprises an ideal WAT. Although previous studies report that esthetics are important for WAT adoption [27,38], the results of this study support this only to a certain degree. Participants recognized the appearance and femininity of the Leaf, but its arduous functionality and perceived inaccuracy devaluated the device. Therefore, although women may appreciate esthetics, it is secondary in importance after comfort and accommodating features.

While discussing the features of the devices, some participants commented about accuracy concerns. Each WAT contains an accelerometer for calculating MVPA, but each uses different criteria to award PA. Participants commented about the various amounts of PA each device awarded for similar routines, leading to a concern about accuracy. This study did not incorporate a gold standard to compare device data with; therefore, the accuracy of devices was not assessed. In addition, accuracy was not the focus of this study but rather the effect that each device had on motivation. Nevertheless, participants’ focus group comments and the large discrepancy between weekly MVPA times and PAVS among Leaf data compared with those of the other devices suggest that the Leaf may deliver the least accurate MVPA measurements. Notably, both the Ring and Watch have been found to have generally acceptable accuracies [39,40], but no published data are available regarding the Leaf.

The use of PAVS and analyzing it based on WAT use is novel to this study. Unfortunately, no statistically significant changes in PAVS occurred among the devices. However, the Watch was the only device with mean PAVS that met the national PA guideline of 150 minutes and trended high from baseline. This, along with the Watch’s motivation ratings and rankings, could suggest that the Watch has positive effect on PA motivation. The Leaf and Ring showed a decrease in PAVS compared with baseline. A long study duration is needed to establish a true association between the devices and the PAVS.

Focus groups resulted in conflicting themes regarding the motivational effects of WATs. Some participants said that the Watch and Ring were motivating, whereas others said that WATs, in general, had no motivational effect. BREQ-2 scores revealed that most women had either high identified or intrinsic regulation at baseline, which shows a pattern toward autonomous PA motivation [30,41]. Participants were already highly motivated, and therefore, WATs may have had little effect on their motivation. These results corroborate another study, which found that current and former users of WATs tend to be inherently motived and, sometimes, doubt device accuracy [42].

### Limitations

We acknowledge several limitations of this study, the first being a small sample size of 15—we had hoped to recruit 25 participants. Our final study population comprised mostly White, middle-class women; therefore, our results are not generalizable to the broad female population. Another limitation of this study was the study period. Owing to time and COVID-19 restrictions, participants wore each WAT for only 1 week, with no washout period between devices. Therefore, our weekly PAVS results may not be an accurate reflection of the WATs’ motivational effectiveness. In addition, 1 week with each device may not fully account for novelty and drop-off effects. The novelty effect is a phenomenon in which the excitement of having a new gadget and the curiosity about its data compels people to use it. The drop-off effect is the gradual disinterest and abandonment of a device as curiosity and enjoyment wear off. In a study, most participants (39/49, 80%) abandoned WATs within the first 2 months [37]. Allowing participants to wear WATs for long periods would help to accurately assess their effects on PAVS and motivation. Future studies should focus on recruiting a diverse population of women, including those with either extrinsic or introjected regulation, which would be beneficial in understanding how WATs affect motivation in the broad female population.

### Conclusions

The findings from this study, a first in evaluating the aspects of WATs that interest women, showed that WATs are generally acceptable to women when they are comfortable to be worn and have appealing features. Workers from a broad array of fields such as health care, fitness, and others who discuss the benefits of PA with their female clients should realize how women view WATs and the role they play in motivation to be active. Exploring the option of WATs with clients can be worthwhile when the women’s perspective is appreciated, for example, by discussing the aspects of trackers that would most likely lead to their adoption and improved motivation when appropriate.

## Abbreviations

BREQ-2: Behavioral Regulation in Exercise Questionnaire–2
MVPA: moderate to vigorous physical activity
PA: physical activity
PAVS: Physical Activity Vital Sign
SDT: self-determination theory
WAT: wearable activity tracker

## Data Availability

The data sets generated or analyzed during this study are available from the corresponding author upon reasonable request.
